# Supplementary material for: Extending Age Ranges in Breast Cancer Screening in Four European Countries: Model Estimations of Harm-to-Benefit Ratios
Source: Cancers (Basel). 2021 Jul 4;13(13):3360. doi: 10.3390/cancers13133360 (PMC8268112; doi:10.3390/cancers13133360)
Supplement: Supplementary file 1 [file cancers-13-03360-s001.zip › cancers-1216671 - Supplementary_final.pdf]

Supplementary Material

A) EU-TOPIA MISCAN-Breast Model Description

1. Model Purpose

Trends in breast cancer incidence and mortality and the (potential) impact of interventions depend on many kinds of factors related to the biology of breast cancer, the characteristics of the population, and the potential impact and usage of early detection and treatment. A simulation model is a helpful tool to estimate the effect of each of the listed factors on cancer incidence and mortality. MISCAN–Breast is developed to analyse trends in breast cancer due to changes in lifestyle, improvement of treatment and implementation of screening strategies. The purpose of MISCAN–Breast can be described in three specific aims:

1. To simulate breast cancer incidence and mortality according to observed figures.
2. To compare screening strategies, allowing the user to improve existing screening programmes as well advising countries on the effects of implementing a breast cancer screening programme.
3. To predict how breast cancer screening and treatment practices will impact future incidence and mortality.

2. Model Overview

2.1. General Model Structure

MISCAN-Breast is a stochastic, semi-Markov microsimulation model. In a microsimulation model, individuals are simulated one at a time instead of as proportions of a cohort. The advantage of this is that new events can be dependent on past events of that individual, giving the model a 'memory'. The model is stochastic, which means that sequences of events are simulated by drawing from distributions of probabilities and durations instead of using fixed values. Therefore, the outcomes of the model are subject to random variation.

MISCAN uses the Monte Carlo method to simulate all events in the program. Possible events are birth and death of a person, onset of a pre-clinical ductal carcinoma in situ (DCIS), transitions between disease states, participation in screening and screen- or clinical detection of a cancer. First, breast cancer incidence and breast cancer mortality are estimated in a situation without screening. Subsequently, screening and treatment related improvements in survival are simulated, in order to determine the impact of screening and treatment on the life histories.

MISCAN–Breast consists of four parts:

- Demography part
- Natural history part
- Treatment part
- Screening part

These parts are not physically separated in the program, but it is useful to consider them separately.

2.2. Demographic Part

MISCAN–Breast first generates a series of individual life histories in the demography part to form a population according to the Demography Parameters. Each woman in the population consists of a date of birth and a date of death from other causes than breast cancer. These dates are drawn from birth and life tables that are representative for the population under consideration. The maximum age that a person can reach in the model is set to 100 years.

2.3. Natural History Part

After individual life histories are simulated in the demography part of MISCAN-Breast, natural histories of breast cancer are simulated for a subset of these women in the Natural History Part (as only a few women will develop breast cancer). Breast cancer starts with the onset of a pre-clinical ductal carcinoma in situ (DCIS) and continues with its progression through the invasive successive states T1A, T1B, T1C and T2+. The development from a DCIS into cancer depends on lymph node status, age-specific transition probabilities and the duration distribution. At each stage, a tumour may become screen-detected if screening is present or clinically detected because of symptoms. The possible transitions between the different states are explained in Figure S1. The life history of each

person is altered according to the breast cancer histories (natural history) that is simulated for that person. If a woman dies from breast cancer before she dies from other causes, her death age is adjusted accordingly.

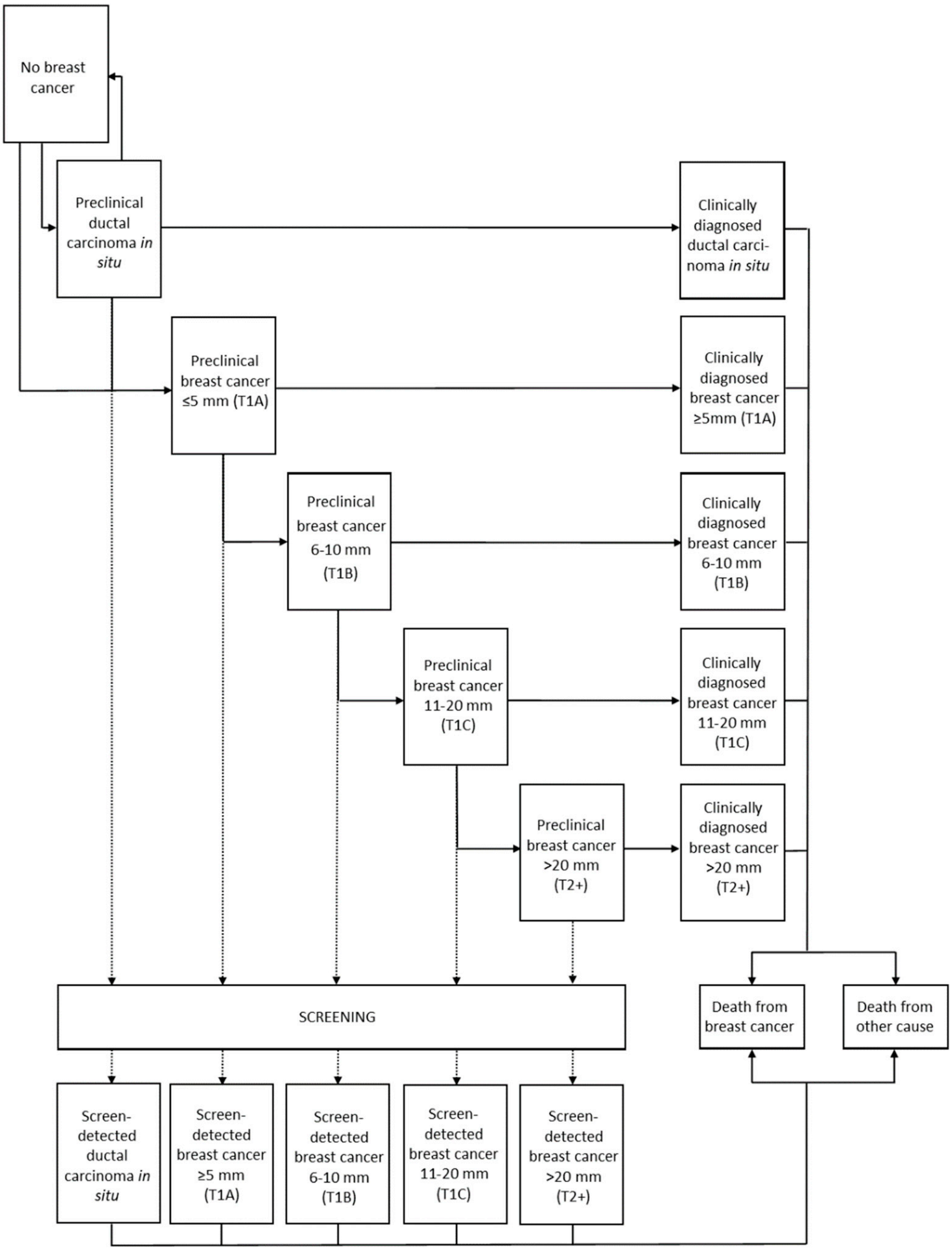

Figure S1. Transitions in the MISCAN-Breast model. The arrows represent the possible transitions.

2.4. Treatment Part

Any screen detected or clinically detected disease transits directly to one of the four treatment states: no therapy; chemotherapy; hormonal therapy; or a combination of chemotherapy and hormone therapy. The probability for a certain treatment is dependent on disease stage, age, calendar year, and detection mode.

A woman in a treatment state can die, either of breast cancer or from other causes. Other-cause mortality is determined by the life table.

The survival time is dependent on age- and stage- and treatment specific survival estimates.

2.5. Screening Part

In the third part of the model, screening for breast cancer is simulated. After the life history of a person is adjusted for breast cancer, the history will now be adjusted for the effects of screening. The screening part is simultaneously run with the natural history part, making detection of DCIS lesions or cancers in different states possible.

Persons can be invited to participate in screening at specified ages as defined in the screening policy. Depending on the test used and the tumour stage at the moment of the screening test, there is a probability of a positive test result. Screening may detect a DCIS lesion or invasive cancers. Women with a true positive result may receive (adjuvant) treatment. In the model, treatment starts immediately once a tumour is screen- or clinically detected. Screening leads to the detection of smaller tumours (in comparison to clinically detected tumours), which may improve survival after diagnosis (stage shift). The effectiveness of screening depends on the screening test characteristics (see Parameter Overview). The effect of screening on the life history of an individual is explained in Figure S2.

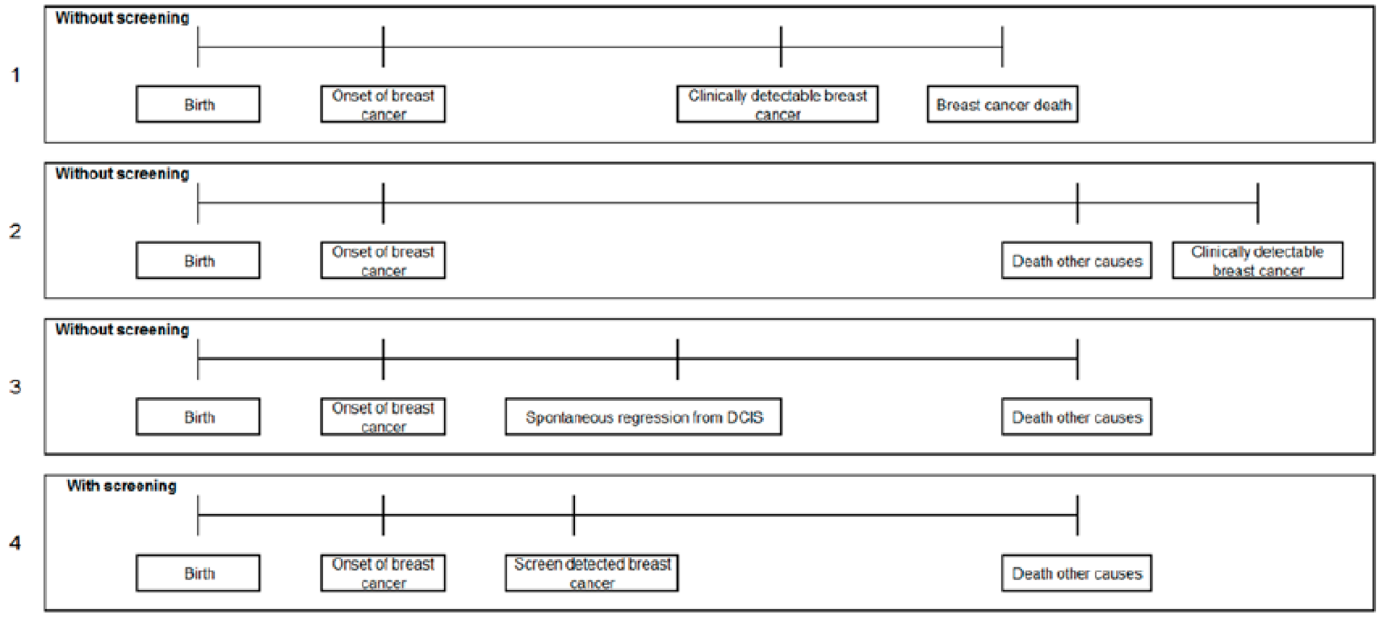

Figure S2. Effect of screening on life history.

Screening affects three women differently. The first box is the life history of a woman who has an onset of breast cancer, is diagnosed clinically, and dies of breast cancer. The second box is the life history of a woman who also has an onset of breast cancer, but who dies of other causes before this would be detected. The third box is the life history of a woman who has an onset of breast cancer, but also a spontaneous regression, this woman would not have been diagnosed without screening. The fourth box indicates the situation for these three women had screening been introduced. The woman in the first box no longer dies from breast cancer; the other two women do not benefit from screening. They have been overdiagnosed.

3. Model Output

All events (for example onset of disease, screen, diagnosis, death by cause) in the model are counted for each woman in two situations: a situation without breast cancer screening and a situation with breast cancer screening. The output of the model is flexible and can be for example: the number of invitations, tests, diagnosis (by mode of detection) and deaths, all by age and year, and when possible cancer stage.

The model can also provide “unobservable” events as lead time, overdiagnosis and breast cancer deaths prevented. In addition, costs and QALYs can be calculated.

4. **Model Assumptions**

As explained in the Section ‘Model overview’, the model consists of four parts; a demography, natural history, a screening and a treatment part. Since some inputs are not (directly) observable in the data and a model is a simplified version of a complex process. Therefore, several assumptions have to be made for the parts.

Because, in principle, all diagnosed breast cancers are treated, it is not exactly known how the disease would have developed without intervention. Hypotheses have to be made on the onset rate of preclinical cancer and the average sojourn time (the period in which a pre-clinical tumour can become detected by screening). The fraction of invasive breast cancers that is preceded by DCIS is uncertain, and it is unknown whether all preclinical DCIS cases progress, or if some are dormant or regress. Furthermore, assumptions need to be made on the age- and stage-specific fatality of cancer and the impact of a screening program. The role of underlying time trends, such as an increasing prevalence of certain risk factors for breast cancer and developments in breast cancer treatment and screening further complicates our analyses.

This does not imply that the natural history of breast cancer is completely unknown. Some indirect inferences can be made from randomized controlled trials and screening data. The average duration of pre-clinical cancer, for instance, is proportional to the ratio between the detection rate in the initial screening round and the clinical incidence rate without screening. Based on stage-specific incidence rates of clinically diagnosed and screen-detected breast cancer and rates of interval cancer, progression and regression rates might be assessed. Using detection rates per screening round and interval cancer rates, the sensitivity of mammography could be estimated. The survival after a breast cancer diagnosis and the influence of a screening program on these survival rates can be deducted from the randomized trials. In MISCAN, the effectiveness of screening was based on the Swedish Trials [1–4]. Despite the fact that these trials have been performed in the seventies and eighties, the observed breast cancer mortality in the Netherlands (and other countries) could be reasonably modelled using these data.

5. **Model Parameters**

5.1. Demographic Part

1. One-year birth cohorts
2. Proportion of the population in each birth cohort
3. Life table

5.2. Natural History Part

1. Mean duration of preclinical screen-detectable cancer by age and stage
2. The probability of a transition between the stages
3. Annual increase in background breast cancer incidence (without screening)
4. Long-term relative survival by clinical stage and age
    a. Without adjuvant treatment
    b. With hormonal treatment
    c. With chemotherapy
    d. With hormonal and chemotherapy
5. Reduction in risk of dying of breast cancer by age and preclinical stage after screen-detection

5.3. Treatment Part

Proportion by age and stage that are treated with adjuvant therapy

5.4. Screening Part

1. Screening attendance by age
2. Test sensitivity of digital mammography by age and preclinical stage
3. Improvement of survival
4. Proportion referral by age

6. **Model Calibration**

The values of some parameters in Section 5 can be observed directly, and will be calculated based on data in the EUTOPIA data template. Other values are available from literature.

However, some model parameters cannot be derived from observational data (e.g., mean duration of preclinical screen-detectable cancer by age and stage). Therefore, these parameter values need to be calibrated (fitted). Calibration involves estimating the parameter values in a way that the simulated outcomes fit the observed data.

6.1. Calibration Parameters

1. Mean duration of screen-detectable preclinical stage by age and stage
2. Test sensitivity of digital mammography by preclinical stage
3. Onset of the disease by age
4. Increasing incidence by year
5. Probabilities of immediate and slow progression from DCIS to T1A
6. Survival

6.2. Regional EU-TOPIA Models

Because the values of the calibrated parameters might differ across Europe, four different models were calibrated. From each European region, an exemplary country with high quality observational data, including the screening behaviour of that population, was selected to be representative for that region (the Netherlands for Western Europe, Finland for Northern Europe, Slovenia for Eastern Europe and Italy for Southern Europe).

6.2.1. Age structure of the female population

Table S1. Age-structure of the exemplary countries, women in 2018.

	% of Total Female Population					
**Country**	**45 to 49**	**50 to 54**	**55 to 59**	**60 to 64**	**65 to 69**	**70 to 74**
Italy	7.9%	8.0%	7.1%	6.3%	6.0%	5.3%
Netherlands	7.3%	7.3%	7.0%	6.3%	5.8%	5.2%
Slovenia	6.8%	7.4%	7.1%	7.1%	6.2%	4.6%
Finland	5.8%	6.6%	6.6%	6.7%	6.9%	5.9%
	% of female population 45–74					
Italy	19.4%	19.7%	17.5%	15.5%	14.7%	13.1%
Netherlands	18.8%	18.9%	18.0%	16.1%	15.0%	13.3%
Slovenia	17.3%	18.8%	18.1%	18.1%	16.0%	11.9%
Finland	15.1%	17.1%	17.1%	17.5%	17.9%	15.3%

Source: https://ec.europa.eu/eurostat/web/population-demography-migration-projections/data/database.

6.2.2. Breast-Cancer Screening Program Netherlands

In the Netherlands, a national population-based screening program is operational since 1990, inviting women 50–69 years of age for a biennial screening examination (Fracheboud et al. 2001). Women 70–75 years of age are invited since 1999. The Dutch Breast Cancer Screening Programme is carried out by five regional Cancer Screening Organisations (65 screening units) and coordinated, monitored and evaluated by the National Institute for Public Health and the Environment. In 2003, the first digital mammography unit was introduced and in 2010, all screening examinations were performed using digital mammography.

The invitation coverage for organized mammography screening was 100.3% in 2016, the participation rate was 77.3%, and the examination coverage was 77.5%.

The detection rate per 1 000 screened women was 1.52 for DCIS and 5.30 for invasive BC in 2016.

6.2.3. Breast-Cancer Screening Program Finland

The nationwide Finnish breast cancer screening program started in 1987 From 1992 until 2006, the national target population consisted of only women aged 50–59 invited biennially, and based on Government Decree on Screenings it widened up to 50–69 during 2007–2016. The coverage of quality assured screening registration has improved with time, and it reached complete coverage of all service providers in 2005. The Mass Screening Registry, a section of the Finnish Cancer Registry, maintains the individual level data on screening invitations to and participation in mammography screening.

The invitation coverage for organized mammography screening was 94.9% in 2014, the participation rate was 82.9%, and the examination coverage was 78.7%.

The detection rate per 1000 screened women was 0.72 for DCIS and 5.36 for invasive BC in 2014.

6.2.4. Breast-Cancer Screening Program Italy

In Italy, screening by mammography is organized regionally and started in the City of Florence in 1990. The current Ministry of Health's guidelines recommend that women aged 50–69 are personally invited every two years. Several regions invite women from the age of 45 (annually) and/or up to age 74–75 (biennially). All 20 regions work under the umbrella of the National centre for screening monitoring (ONS). Together with the Italian group for mammography screening (GISMa), ONS is responsible for monitoring, performance evaluation, data collection and promotion of the organized breast screening programmes in Italy.

The invitation coverage for organized mammography screening was 81.0% in 2015, the participation rate was 54%, and the examination coverage was 43.8%. We assume that 19% of all woman age 50–69 were screened outside the programme.

The detection rate per 1000 screened women was 0.7 for DCIS and 3.7 for invasive BC in 2013.

6.2.5. Breast-Cancer Screening Program Slovenia

The breast cancer screening programme in Slovenia (DORA) is organized, national and population based. The implementation started in 2008 and has been gradually expanded until rollout was completed in April 2018. DORA invites women aged 50 to 69 years to screening mammography every two years. In 2016 screenings were carried out in 11 screening units; on 12 stationary screening mammographs and in 2 mobile screening units. Responsible institution: Institute of Oncology Ljubljana.

The invitation coverage for organized mammography screening was 53.4% in 2016, the participation rate was 75.7%, and the examination coverage was 40.4%. We assume that 13% of all woman age 50–69 were screened outside the programme.

The detection rate per 1000 screened women was 1.31 for DCIS and 5.03 for invasive BC in 2016

6.3. Calibration Process

In calibrating each new country-specific MISCAN-Breast model, we used a specific calibration process composed of 4 steps (Figure S3):

1. The starting point is an existing model that was calibrated for the Netherlands (Model A)
2. All model inputs that are based on observed data, such as the demographic characteristics of the population, cancer survival and the implemented screening programme, are adjusted to represent the situation in country B (step II).
3. Observed data is collected that will be compared with the model outputs. These observed data are the so-called calibration targets: BC incidence and mortality rates, interval cancer rates, stage distribution and detection rates (Step III).
4. Based on the observed data the model optimizes a set of unobservable parameters to meet the observed data in the model output (step IV). MISCAN uses the Nelder and Mead simplex ("Amoeba") multivariate minimization routine, which has been adapted for optimizing random functions. The model runs repeatedly, and the simulation runs are compared to the counts in the observations. The total deviance is the sum of the individual deviances. A convergence criterion is set by a Kendall Tau test. The required significance level of the test can be specified.

5. After the model fitted a set of unobservable parameters, the outcomes of step IV are evaluated. First by evaluating the model fit with the calibration targets (by visual inspection including 95% confidence interval). If the fit is not satisfying, the model inputs are re-evaluated and step IV is repeated. Parameters of the natural history of the cancer, e.g. stage-specific mean duration of screen-detectable breast cancer and progression, are initially assumed to be equal between model country A and B. Only if the calibrated model for country B does not meet the calibration targets, these natural history parameters will be calibrated as well, together with the initial set of calibration parameters.
6. If the fit is satisfying, the model is validated by replicating mortality reduction due to mammography screening based on outcomes observed in the selected published studies (data not used in the calibration process, external validation). Briefly, we adjust the models to reflect specific demographics (e.g. year and age of BC deaths),and screening policy of this study (starting age, stopping age, screening modality, interval, participation) in accordance with the selected published study for country B. Then we compare the model predictions and the observed outcomes of the study. If the outcomes predicted by the model are within the 95% confidence interval of the corresponding study's observed outcomes, a calibrated and validated model for country B is ready to be used.

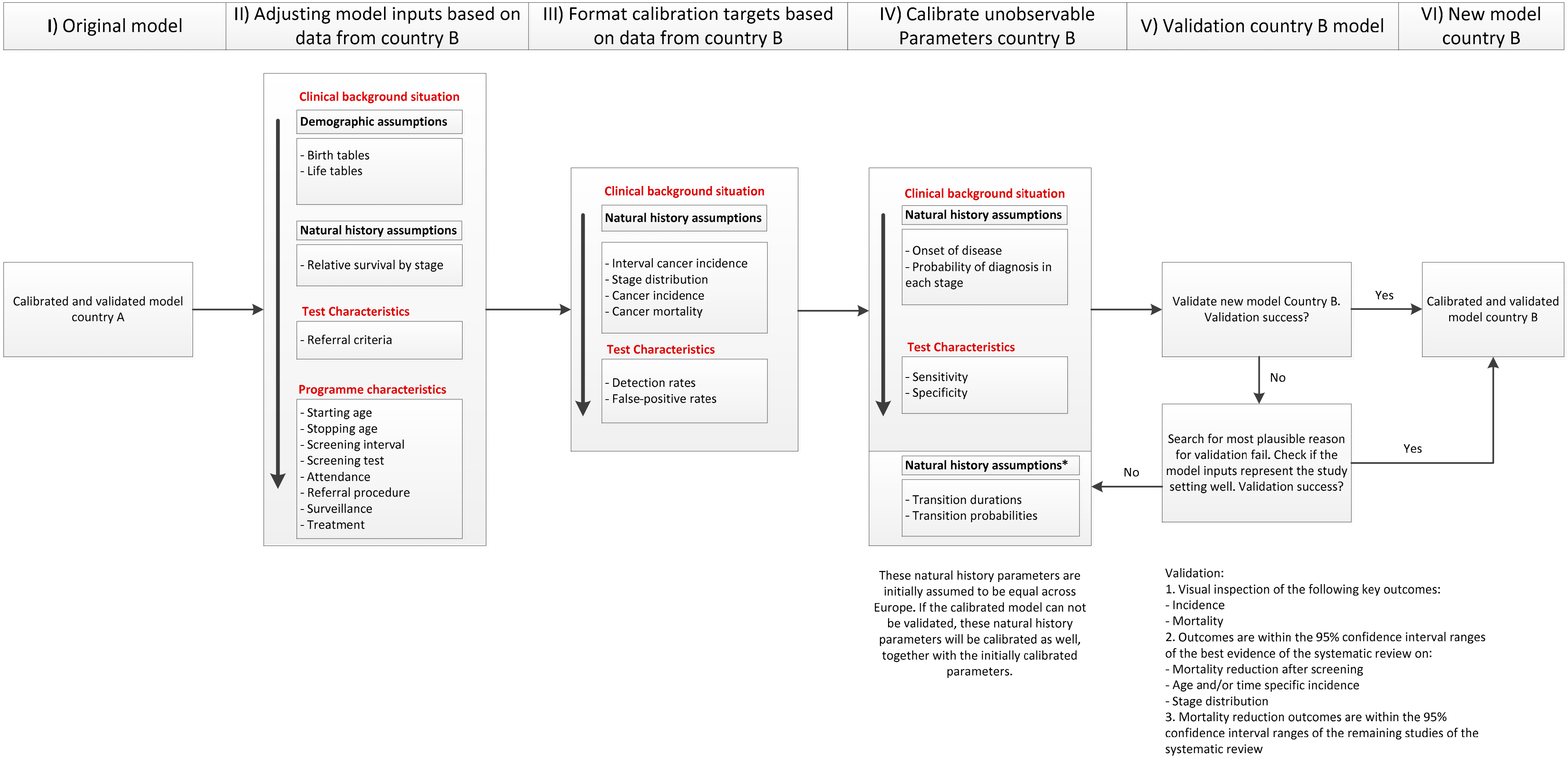

Figure S3. Calibration and validation process for development of MISCAN-Breast country specific models.

6.4. *Calibration Results*

6.4.1. Country Specific Calibration Parameters

Table S2. Model input parameters.

Input parameter	Netherlands	Finland	Italy	Slovenia
Stage-specific sensitivity of digital mammography DCIS	0.865	0.596	0.821	0.726
Stage-specific sensitivity of digital mammography T1a	0.553	0.811	1.000	0.785
Stage-specific sensitivity of digital mammography T1b	0.481	0.761	0.717	0.656
Stage-specific sensitivity of digital mammography T1c	0.857	0.946	0.814	0.780
Stage-specific sensitivity of digital mammography T2+	1	1	1	1
Breast cancer onset	0.291	0.279	0.267	0.267
Onset hazard age 30 years	0.0000705	0.0000135	0.000000302	0.0000201
Onset hazard age 50 years	0.0098	0.0067	0.0078	0.0060
Onset hazard age 70 years	0.0190	0.0135	0.0148	0.0060
Onset hazard age 100 years	0.0245	0.0000242	0.0018	0.0000158
Stage-specific duration (years) screen-detectable preclinical stage DCIS	0.669	0.921	0.669*	1.525
Stage-specific duration (years) screen-detectable preclinical stage T1a	0.720	1.079	0.720*	0.702
Stage-specific duration (years) screen-detectable preclinical stage T1b	1.106	1.550	1.106*	1.495
Stage-specific duration (years) screen-detectable preclinical stage T1c	1.492	1.200	1.492*	1.737
Stage-specific duration (years) screen-detectable preclinical stage T2+	1.171	1.269	1.171*	1.304
Mortality factor ages 0–72.5	2.2	0.8	2	1.2
Mortality factor ages 72.5–100	4.5	5	5	5
Referral rate <50	0.030	0.030	0.065	0.040
Referral rate >50	0.023	0.028	0.058	0.034

* This natural history parameter is assumed to be the same as in Model A (Netherlands) as the calibration results were satisfying.

6.5. The Netherlands (West, model A)

The MISCAN model for breast cancer screening in the Netherlands has been well reported and validated in the past [5,6]. The parameters were calibrated to recent data from the Dutch screening organizations on interval cancers (between 2004 and 2011), screen-detected cancers (2004–2013) and stage distribution at detection for screen-detected cancers and interval cancers. Simultaneously, the parameters were calibrated to data on breast cancer incidence between 1975 and 2013 by five year age groups, from the National Cancer Registry and the Eindhoven Cancer Registry.

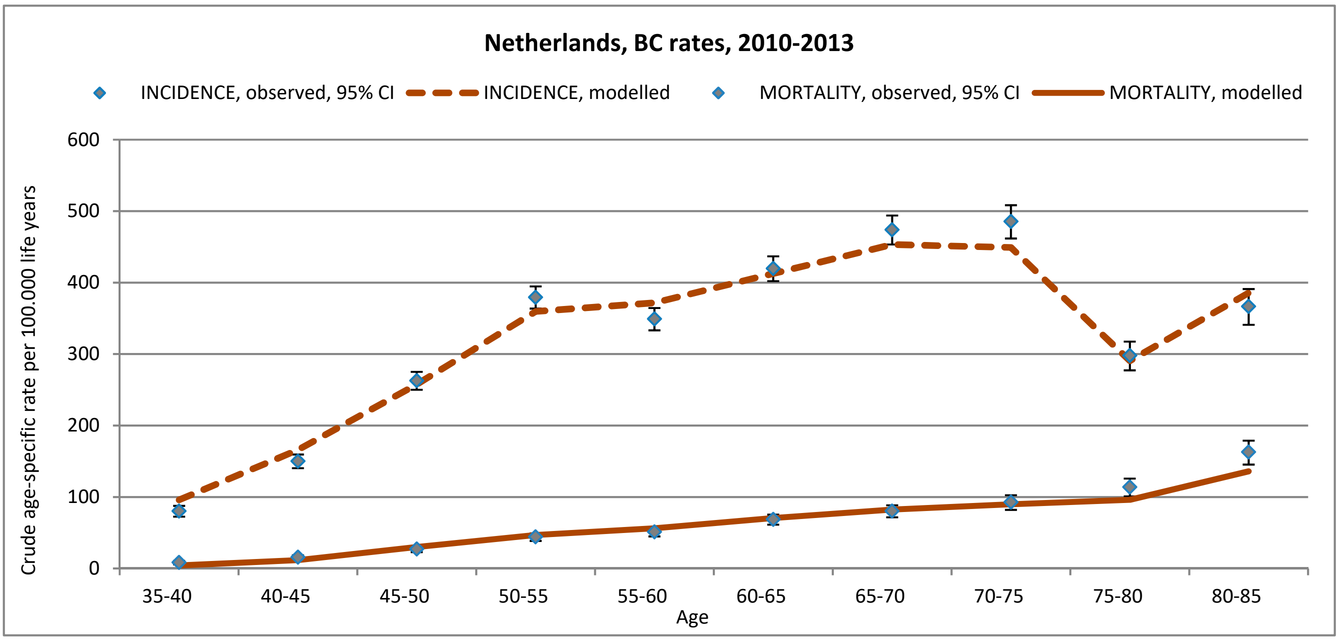

Figure S4. Fit of the model predictions with observed breast cancer incidence and mortality in the Netherlands, 2010–2013.

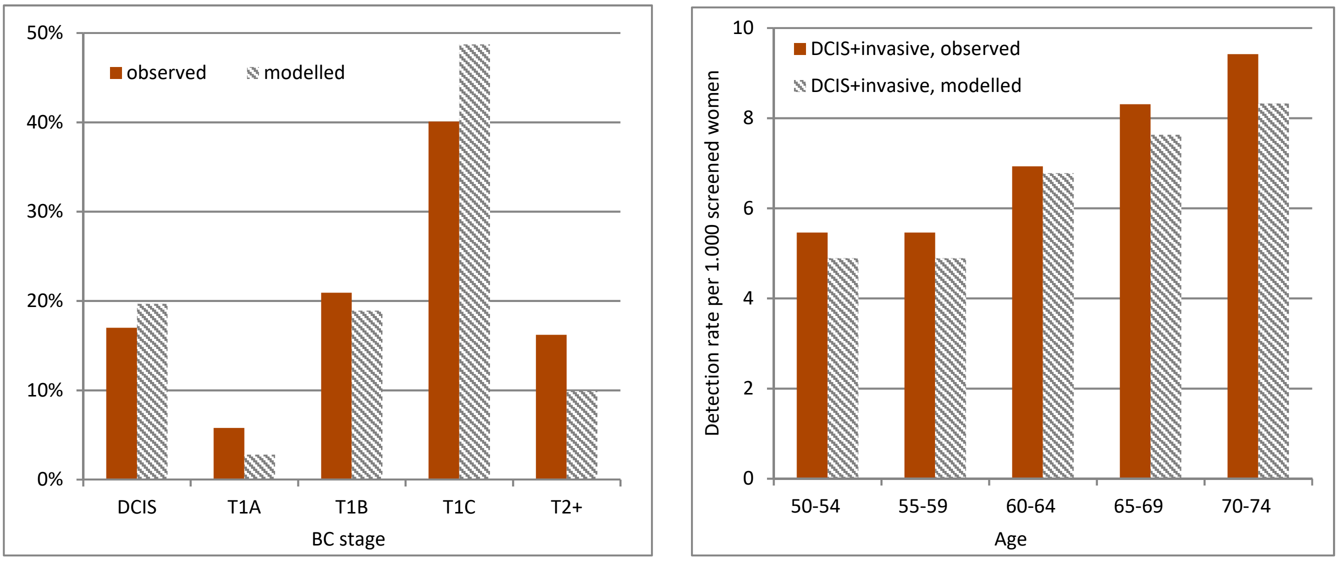

Figure S5. Fit of the model predictions with observed stage distribution (left, only screen detected cancers, 2010–2014) and detection rate (right, 2013) in the Netherlands.

6.6. Finland (North)

For the MISCAN model for breast cancer screening in Finland we modelled the female Finnish population born 1910–2016. As direct model inputs we included the screening histories between 1987 until 2016 [7,8]. We calibrated age-specific and period-specific incidence hazards and levels, DCIS probabilities, durations and sensitivity. As the observed data was not conform the TNM stage classification, the BC stage distribution data was adjusted as follows: DCIS, localized (including T1A-T2+, node negative) and non—localized (including T1A-T2+, node positive). We fitted the Finnish model on age-specific breast cancer incidence in two periods simultaneously: 1975–1985 and 1995–2014 (data from the Finnish Cancer Registry).

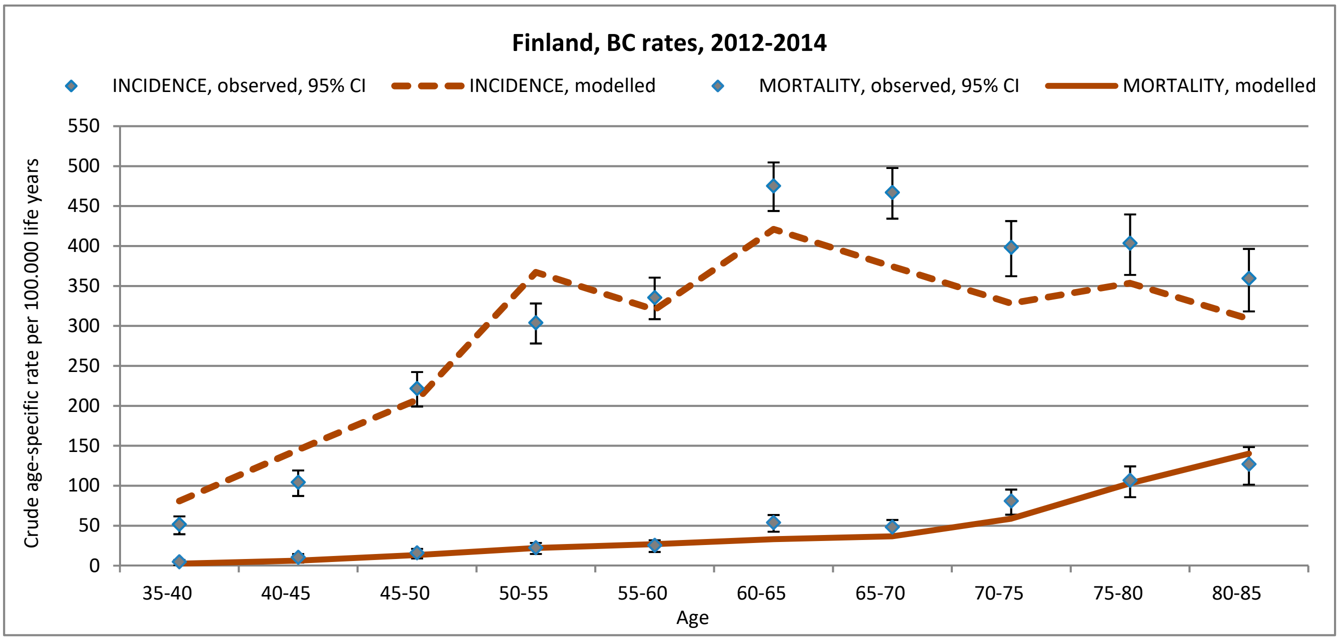

Figure S6. Fit of the model predictions with observed breast cancer incidence and mortality in Finland, 2012–2014.

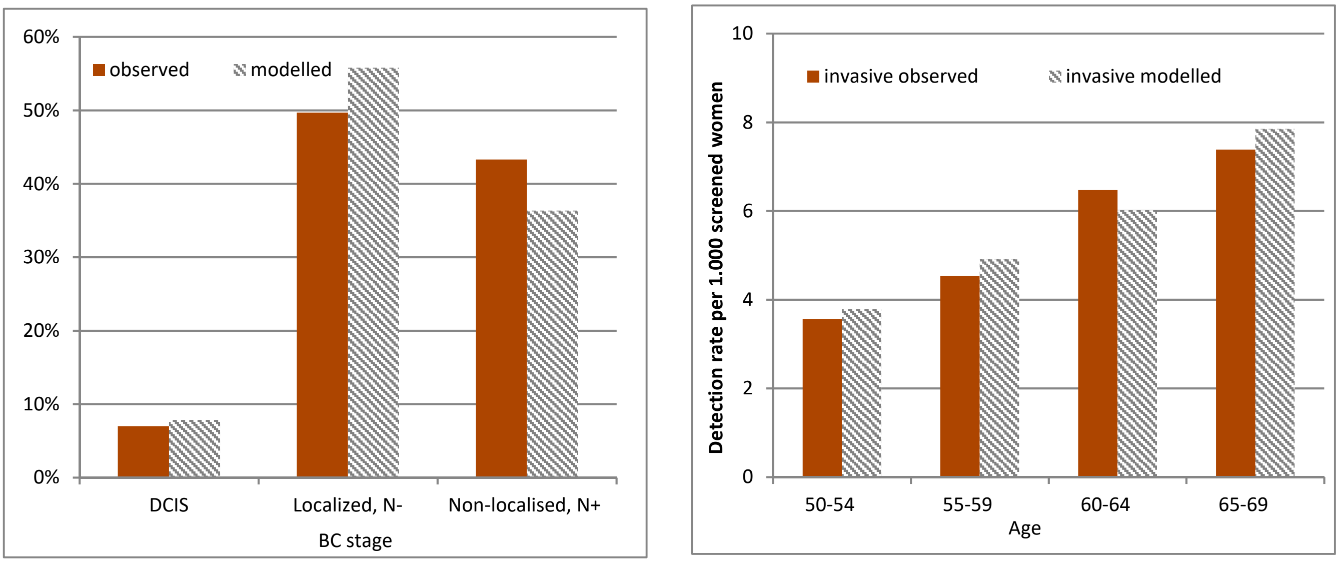

Figure S7. Fit of the model predictions with observed stage distribution (left, only screen detected cancers, 2006–2011) and detection rate (right, 2013) in Finland.

6.7. Italy (South)

The Italian MISCAN Breast model was calibrated on age-specific and stage-specific breast cancer incidence and mortality in Italy in 2006-2009 with data from the Italian Association of Cancer Registries (AIRTUM), which covers approximately one third of the total Italian female population. Stage distribution parameters were calibrated using data from the Cancer Screening National Monitoring reports. We modelled the age distribution of the female Italian population born between 1920 and 1980 using data from the Human Mortality Databases.

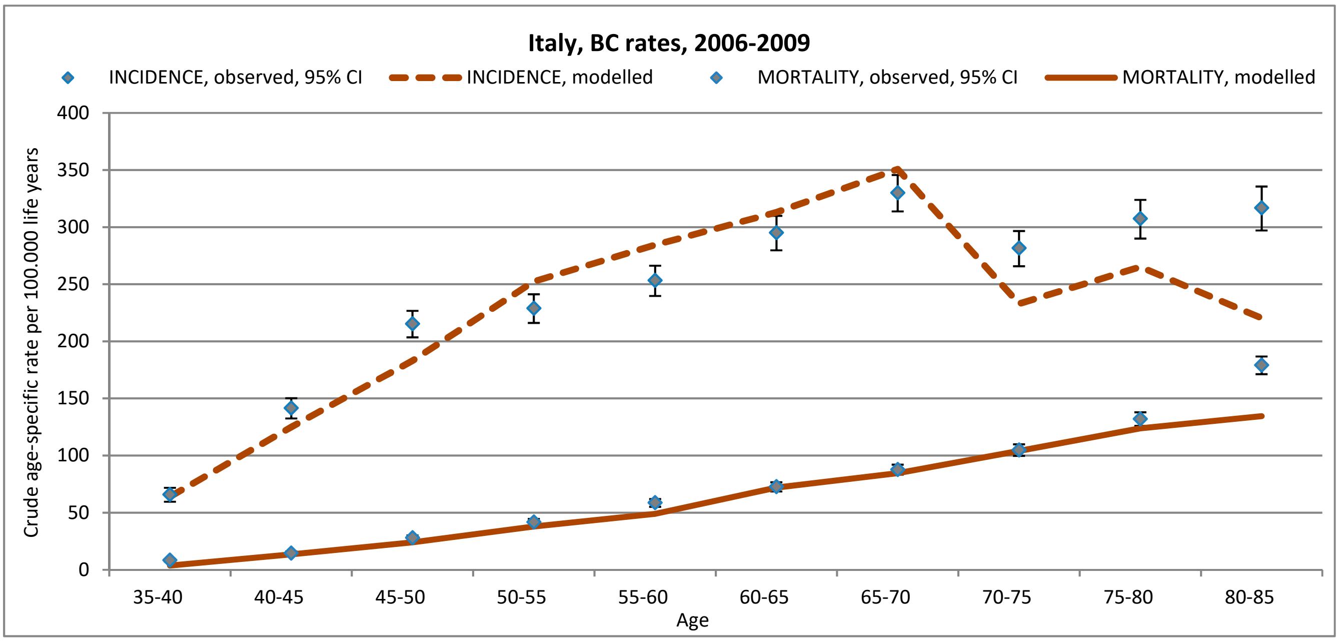

Figure S8. Fit of the model predictions with observed breast cancer incidence and mortality in Italy, 2006–2009.

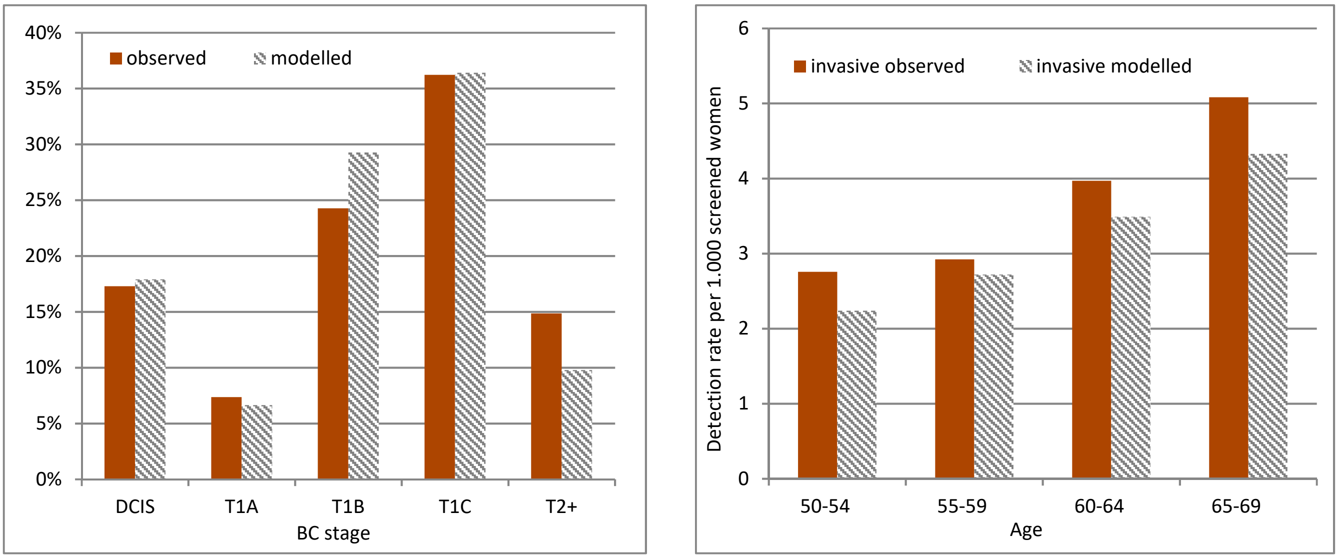

Figure S9. Fit of the model predictions with observed stage distribution (left, only screen detected cancers) and detection rate (right) in Italy, 2013.

6.8. Slovenia (East)

The Slovenian MISCAN Breast model was calibrated on age-specific breast cancer incidence in 1975–2014 (CR of Slovenia). As the implementation of the breast cancer screening program in Slovenia (DORA) started in 2008, the estimate of total coverage (opportunistic and organized) was mostly based on expert opinion for 1990–2008. We modelled the female Slovenian population born 1926–1982 using data from the Human Mortality Databases. We validated the Slovenian model by comparing the observed and modelled Interval cancer rates in 2014

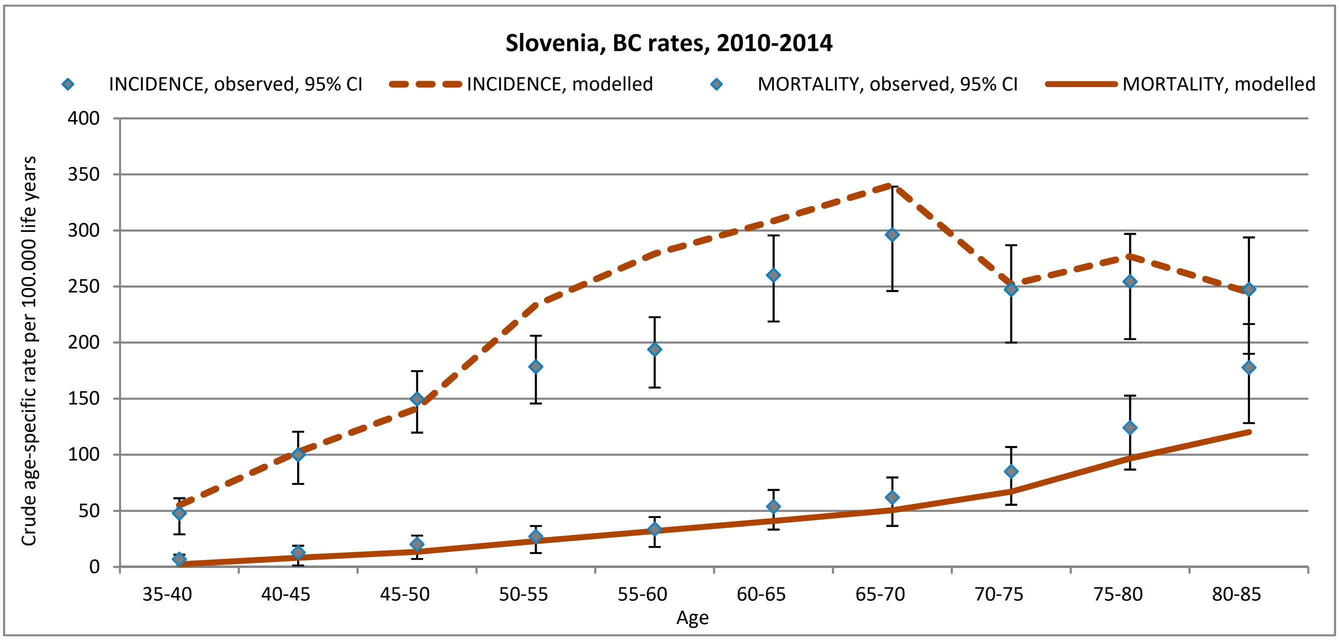

Figure S10. Fit of the model predictions with observed breast cancer incidence and mortality in Slovenia, 2010–2014.

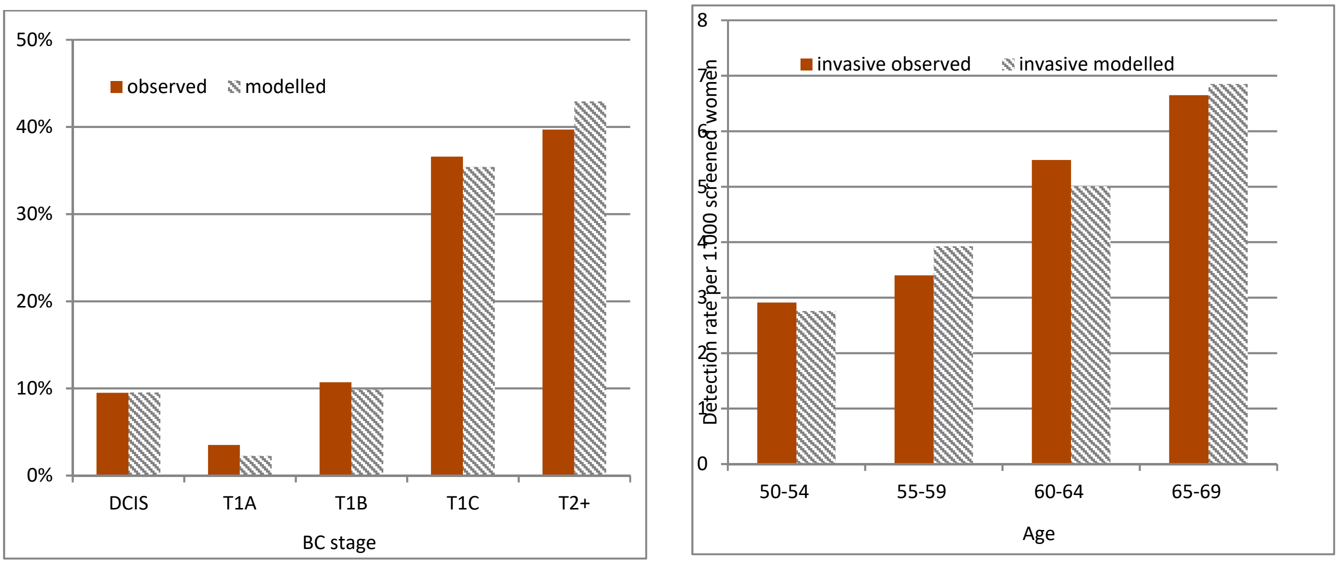

Figure S11. Fit of the model predictions with observed stage distribution (left, only screen detected cancers, 2011–2015) and detection rate (right, 2013) in Slovenia.

7. Model Validation

As the EU-TOPIA MISCAN-Breast model is also used to inform policy makers, it is important to give insight in the calibration (model fitting) methods and the accuracy of model outcomes [9,10]. The latter can be achieved by providing transparency, i.e. reporting on the structure of the model, the value of parameters and the rationale for the assumptions used (provided through the companion MISCAN-Breast model description), and by describing how the model has been validated.

To ensure the validity of our calibrated models and consequently the usefulness in predicting, monitoring, and improving the existing screening programs, it is very important to validate our model predictions against observed data. With this aim: i) a systematic review was performed to summarize the evidence for breast cancer specific mortality reduction due to screening in Europe[11]; ii) some studies ("best evidence") were identified and selected for validating the fully calibrated models for: Finland, Netherlands, Italy and Slovenia. These countries are exemplary for the Northern, Western, Southern and Eastern regions of Europe.

7.1. Best Evidence

7.1.1. Definition "Best Evidence"

As an extension of the systematic reviews, we identified those among all included studies in the review, that provides "best evidence " in observed data which the MISCAN model output can be compared to. We defined the best evidence study, judging the level of evidence of each study based on a group of factors. Those had a different hierarchic order and were reported in order of importance as follow:

1. Country was the strongest factor for this report as we looked for best data to validate models for exemplary countries.
2. Outcome prioritizes cancer specific mortality for all three cancer sites. Alternatively, incidence is a useful outcome to validate MISCAN-Colon models rated as Level II (Studies that assessed the effect of screening on CRC incidence reduction).
3. Study setting reflects the favoritism of data from actual cancer screening programs over other study settings, including randomized controlled trials and observational studies.
4. Study design/Risk of bias is a combination of study design and risk of bias and is based on the results of the prior quality assessment.

Table S3. List of factors forming the judgement of level of evidence of each study.

Criteria	Level	Type of Studies Retrieved
Country	I	Studies conducted in that specific country (national level).
	II	Studies conducted inside that specific country (regional level).
	III	Studies conducted in neighbouring countries within the same European region (national level).
	IV	Studies conducted in neighbouring countries within the same European region (regional level).
Outcome	I	Studies that assessed the effect of screening on cancer specific mortality reduction.
	II	Studies that assessed the effect of screening on cancer specific incidence reduction.
	III	Studies that assessed the effect of screening on overall mortality reduction.
Study setting	I	Screening program evaluation study.
	II	Research study.
Study design /Risk of bias	I	Randomized Control Trials with Low Risk.
	II	Randomized Control Trials with Moderate Risk / Observational studies with Low Risk (score of 8 or 9);
	III	Randomized Control Trials with High Risk / Observational studies with Moderate Risk (score 5 to 7).
	IV	Observational studies with High Risk (score from 0 to 4).

Thus, a study conducting a screening program evaluation within an exemplary country and investigating the impact of screening on cancer specific mortality will be considered as highest level of evidence. However, when no study will show all these factors at the same time the selection will be performed giving priority, respectively, to country, outcome, study setting, and combination of study design and risk of bias.

7.1.2. Best Evidence per European Region

No studies from Eastern Europe met the initial inclusion criteria and subsequently qualified as "best evidence".

Table S4. Best evidence for 3 of the 4 European countries and their respective point estimates on breast cancer mortality reduction due to mammography screening.

Study	Region	Country	Study Type	Target Age	Effect Sizes for Breast cCancer Mortality [1], (95%CI)
Heinävaara S, 2016 [7]	North	Finland	Case-control	50–69	HR = 0.67 (0.49–0.90)
Puliti D, 2008 [12]	South	Italy	Case-control	50–74	OR = 0.50 (0.42–0.60)
Paap E, 2014 [13]	West	Netherlands	Case-control	50–75	OR = 0.42 (0.33–0.53)

[1] Attenders/non-attenders. CI = Confidence interval, HR = Hazard Ratio, OR = Odds ratio CC = Case Control study, OR = Odds ratio, HR = Hazard Ratio, CI = Confidence interval.

7.2. Validation Results

7.2.1. The Netherlands

In a multi-region case-referent study, Paap [13] included breast cancer deaths in women aged 50–75 between 2004 and 2005 and estimated the benefit of the population-based screening program on breast cancer specific mortality to be as high as 58% (adjusted OR = 0.42; 95% CI 0.33-0.53) for screened compared to unscreened women.

The study included five of the nine regional screening organisations (which cover more than half of the target population for screening in the Netherlands): Stichting Bevolkingsonderzoek Noord-Nederland (BBNN), Stichting Kankerpreventie IKA, Stichting Kankerpreventie en screening Limburg (SKsL), Stichting Bevolkingsonderzoek Borstkanker Zuidwest Nederland (SBBZWN), and Stichting Vroege Opsporing Kanker Oost-Nederland (SVOKON).

Table S5. Estimates of breast cancer mortality reduction in Dutch best evidence [13] and modelled estimate for the same period of time and age group, screened vs. un-screened women.

Study	Age Group	Study Period	Study Estimates		Model Estimate [a]	
			Odds Ratio (95% CI)*	BC Mortality Reduction	Odds Ratio (95% CI) *	BC Mortality Reduction
Paap et al. 2014	50–75	2004–2005	**Pooled 0.42 (0.33–0.53)** BBNN 0.40 (0.22–0.74) IKA 0.38 (0.25–0.57) SKsL 0.24 (0.10-0.62) SBBZWN 0.49 (0.30–0.78) SVOKON 0.51 (0.30–0.87)	58%		43.5%

[a] 100% Attendance to screening assumed. * Estimates with correction for self-selection bias. Abbreviations: confidence interval (CI); breast cancer (BC).

Our estimate of 43.5% (odds ratio of 0.57) breast cancer mortality reduction due to screening with 100% attendance to screening lies outside of the confidence interval of the pooled odds ratio in the study. However, the model estimate is within the confidence interval of each individual region.

The estimates of reduction in breast cancer mortality of the Dutch best evidence could not be reproduced well, as the model estimates were considerably lower. The model estimates do therefore not overestimate the screening effect, as has been argued by critics, and may even be conservative.

7.2.2. Finland

Heinävaara [7] evaluated the long-term effect of organized mammography screening on incidence based mortality (IBM) in Finland in 1992–2011 among 50–84-year-old women using a case–control design with non-restrictive eligibility criteria of controls. Organised screening decreases mortality from breast cancer by 33% in women attending screening (HR = 0.67 [0.49–0.90], corrected for self-selection bias).

Table S6. Estimates of breast cancer mortality reduction in Finish best evidence[7] and modelled estimate for the same period of time and age group, screened vs. un-screened women.

Study	Age Group	Study Period	Study Estimates		Model Estimate [a]	
			Hazard Ratio (95% CI)	BC Mortality Reduction	Hazard Ratio (95% CI) *	BC Mortality Reduction
Heinävaara et al. 2016	50–84	1992–2011	0.67 (0.49–0.90)	33%	0.77	22%

[a] 100% Attendance to screening assumed. * Estimates with correction for self-selection bias. Abbreviations: confidence interval (CI); breast cancer (BC).

The simulation of the calibrated Finish MISCAN model could replicate the estimate for breast cancer specific mortality reduction due to screening. The model results underestimate the effect of BC screening on mortality reduction, but it is within the confidence interval of the estimates from Heinävaara et al.

7.2.3. Italy

The aim of the case–control study of Pulity [12] has been to evaluate the effectiveness of service screening programmes in reducing breast cancer mortality in the Italian areas participating in the IMPACT study (Piedmont, Tuscany, Umbria, Veneto, Emilia-Romagna). For women between 50 and 74 years who died between screening activation and 2002, the odds ratio comparing screened with unscreened women was 0.50 (95% CI: 0.42–0.59).

Table S7. Estimates of breast cancer mortality reduction in Italian best evidence [12] and modelled estimate for the same period of time and age group, screened vs. un-screened women.

Study	Age Group	Study Period	Study estimates		Model estimate [a]	
			Odds Ratio (95% CI)	BC Mortality Reduction	Odds Ratio (95% CI)*	BC Mortality Reduction
Puliti et al. 2008	50-74	1990-2002	**0.50 (0.42–0.60)**	50%	0.44	56%

[a] 100% Attendance to screening assumed. * Estimates with correction for self-selection bias. Abbreviations: confidence interval (CI); breast cancer (BC).

The estimate for breast cancer specific mortality reduction due to screening in Italy could be replicated with the simulation of the calibrated Italian MISCAN model. The model results overestimate the effect of BC screening on mortality reduction, but it is within the confidence interval of the estimates from Puliti et al.

7.2.4. Slovenia

Due to the lack of best evidence from Eastern Europe, we alternatively tried to validate the Slovenian model by comparing the observed and modelled Interval cancer rates–provided by the EU-TOPIA consortium members. Per 5 year age-group, we modelled the clinically detected cancers in 2014, detected 12 and 24 months after the last negative screen (first/subsequent).

Table S8. Observed interval cancer rate in Slovenia and modelled estimate for the same period of time and age group.

		**Interval Cancer Rate per 1000 Visits**			
		Diagnosed within the first year after test		Diagnosed within the second year after test	
		observed	modelled	observed	modelled
Initial[1]	50–54	0.32	0.76	1.29	0.89
	55–59	0	0.49	1.68	0.84
	60–64	1.08	0.69	0.36	1.18
	65–69	0	0.20	1.65	0.60
Subsequent[2]	50–54	0.73	0.31	0.24	0.52
	55–59	0.19	0.48	1.14	0.75

60–64	0.38	0.57	0.95	0.79
65–69	0.71	0.36	0.95	0.79

[1] Initial screening is the first screening examination of women within the screening programme, regardless of the organisational screening round in which the examination takes place. [2] Subsequent screening includes all screening examinations of women within the screening programme following an initial screening examination, regardless of the organisational screening round in which the examination takes place.

Keeping in mind that interval cancers are based on rather small numbers, we are satisfied with the results.

B) Results of the Sensitivity analysis

Table S9. Harms-to-benefit-ratios in response to variation in input parameters, per country and screening strategy.

		Harm-to-Benefit-Ratios																			
		Overdiagnosed BC cases/BC Deaths Averted					False-Positives/BC Deaths Averted					Overdiagnosed BC Cases/LY Gained					False-Positives/LY Gained				
**Country**	**Screening strategy**	**Sens Highest**	**Sens Lowest**	**Referral Highest**	**Referral Lowest**	**Cover Age**	**Sens Highest**	**Sens Lowest**	**Referral Highest**	**Referral Lowest**	**Cover Age**	**Sens Highest**	**Sens Lowest**	**Referral Highest**	**Referral Lowest**	**Cover Age**	**Sens Highest**	**Sens Lowest**	**Referral Highest**	**Referral Lowest**	**Cover Age**
**Slovenia**	45–74	0.51	0.52	0.52	0.52	0.55	43.36	49.97	85.01	36.38	38.34	0.04	0.04	0.04	0.04	0.04	3.30	3.82	6.50	2.78	2.96
	45–69	0.41	0.43	0.43	0.43	0.45	45.25	52.69	89.44	38.48	41.55	0.03	0.03	0.03	0.03	0.03	3.16	3.69	6.26	2.69	2.93
	50–74	0.54	0.55	0.55	0.55	0.58	31.49	36.32	66.14	23.01	27.65	0.04	0.04	0.04	0.04	0.05	2.57	2.98	5.43	1.89	2.28
	50–69*	0.44	0.45	0.45	0.45	0.48	32.26	37.65	68.44	23.97	29.52	0.03	0.03	0.03	0.03	0.04	2.43	2.83	5.16	1.81	2.23
**Finland**	45–74	0.40	0.41	0.38	0.38	0.38	31.19	36.95	75.70	31.92	29.20	0.03	0.03	0.03	0.03	0.03	2.36	2.79	5.72	2.41	2.20
	45–69	0.35	0.41	0.32	0.32	0.32	34.17	36.95	82.98	35.16	32.66	0.02	0.03	0.02	0.02	0.02	2.33	2.79	5.65	2.40	2.22
	50–74	0.54	0.53	0.51	0.51	0.50	25.57	29.16	59.40	20.59	23.67	0.04	0.04	0.04	0.04	0.04	2.07	2.36	4.80	1.67	1.90
	50–69*	0.36	0.33	0.34	0.34	0.34	26.88	30.51	63.39	21.65	25.47	0.03	0.02	0.02	0.02	0.02	1.96	2.23	4.63	1.58	1.85
**Netherlands**	45–74	0.30	0.28	0.30	0.30	0.30	16.79	19.17	45.86	18.78	16.22	0.02	0.02	0.02	0.02	0.02	1.18	1.35	3.21	1.32	1.14
	45–69	0.23	0.21	0.23	0.23	0.24	16.89	19.41	46.03	18.99	16.59	0.02	0.01	0.02	0.02	0.02	1.11	1.28	3.01	1.24	1.09
	50–74*	0.32	0.30	0.32	0.32	0.32	10.29	11.90	36.26	11.64	9.80	0.02	0.02	0.02	0.02	0.02	0.77	0.90	2.73	0.87	0.74
	50–69	0.25	0.23	0.25	0.25	0.26	10.16	11.84	35.72	11.57	9.91	0.02	0.02	0.02	0.02	0.02	0.71	0.83	2.50	0.81	0.70
**Italy**	45–74	0.28	0.26	0.28	0.28	0.30	54.97	61.45	58.23	24.59	47.29	0.02	0.02	0.02	0.02	0.02	3.87	4.35	4.11	1.74	3.40
	45–69	0.21	0.19	0.22	0.22	0.23	55.32	62.34	58.69	24.90	48.46	0.01	0.01	0.01	0.01	0.02	3.61	4.09	3.84	1.63	3.22
	50–74	0.29	0.28	0.30	0.30	0.32	43.68	48.70	46.29	15.74	37.46	0.02	0.02	0.02	0.02	0.02	3.30	3.69	3.51	1.19	2.89
	50–69*	0.23	0.21	0.23	0.23	0.24	43.08	48.46	45.73	15.63	37.63	0.02	0.01	0.02	0.02	0.02	3.02	3.40	3.21	1.10	2.69

Sens highest: application of the highest values for stage-specific sensitivity across all countries; Sens lowest: application of the lowest values for stage-specific sensitivity across all countries; Referral highest: application of the highest referral rates across all countries; Referral lowest: application of the lowest referral rates across all countries. Attendance: application of observed attendance; * Current screening strategy. BC: breast cancer; LY: Life years.

The figures S12–16 contain—per country—the results of the sensitivity analysis for the reference age-group 50–69. For each of the four ratios, the bars present the percentage changes in response to variations in input parameters, compared to the base analysis. These variations include application of observed examination coverage from each country (striped bar), and application of the highest values for stage-specific sensitivity and referral rates (dotted bars) and the lowest values for stage-specific sensitivity and referral rates (blue bars). OD: Overdiagnosis; BC: Breast cancer; FP: False positive results; LY: Life years

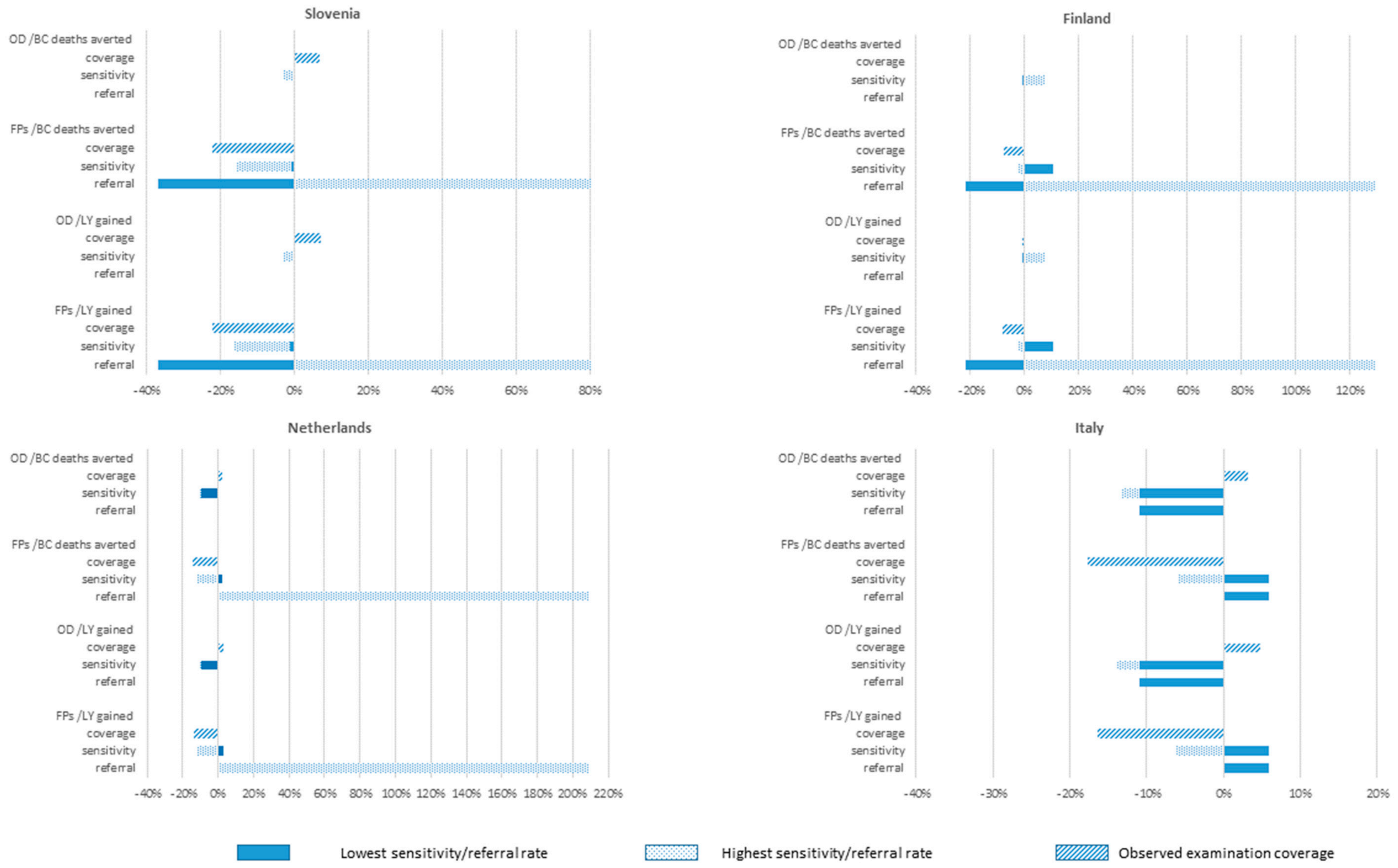

Figure S12. Percentage change in harms-to-benefit-ratios in response to variation in input parameters, per country, age-group 50–69.

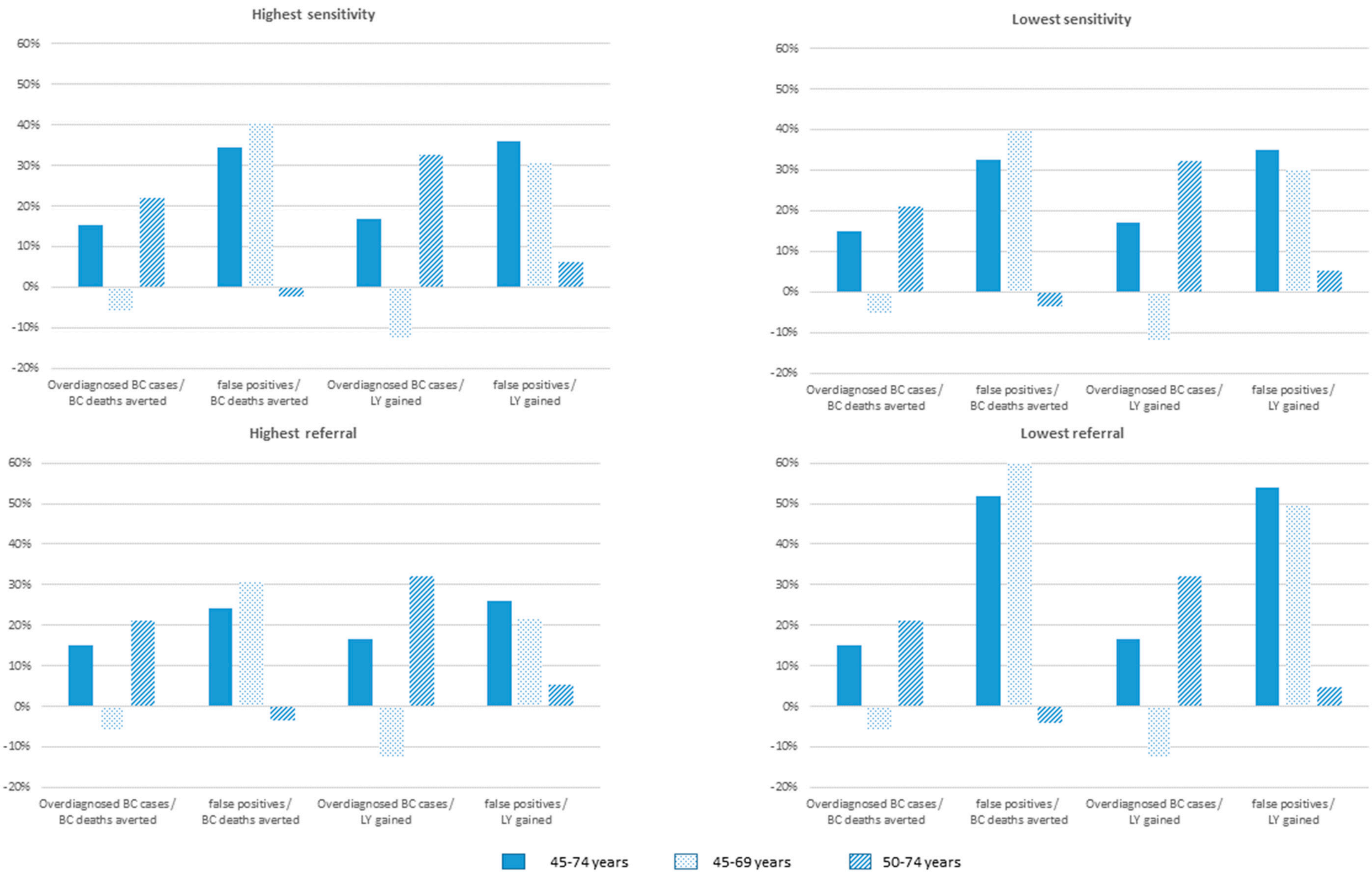
Highest sensitivity
Lowest sensitivity
Highest referral
Lowest referral
60%
50%
40%
30%
20%
10%
0%
-10%
-20%
Overdiagnosed BC cases / BC deaths averted
false positives / BC deaths averted
Overdiagnosed BC cases / LY gained
false positives / LY gained
45-74 years
45-69 years
50-74 years

Figure S13. *Cont.*

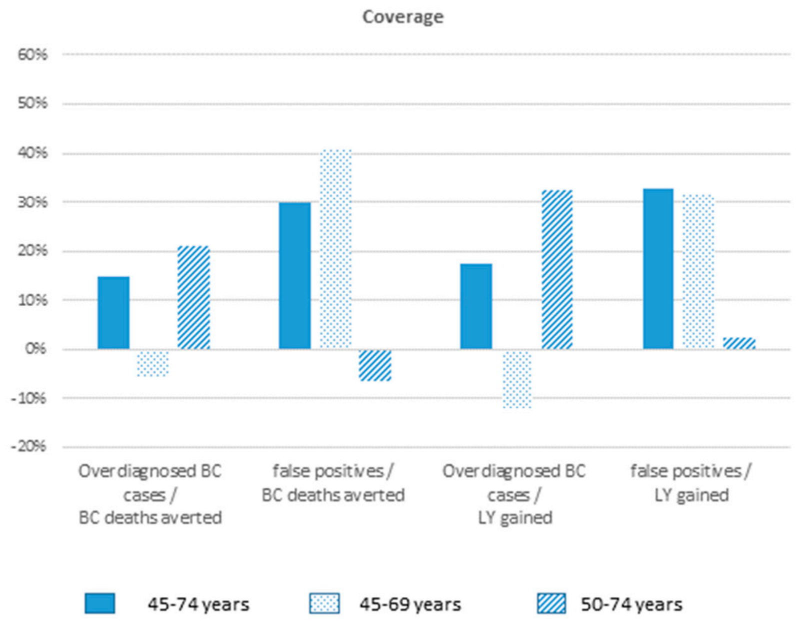

Figure S13. Percentage change in harms-to-benefit-ratios in comparison to the reference age-group 50–69, per screening scenario and varied parameter of the sensitivity analysis. SLOVENIA.

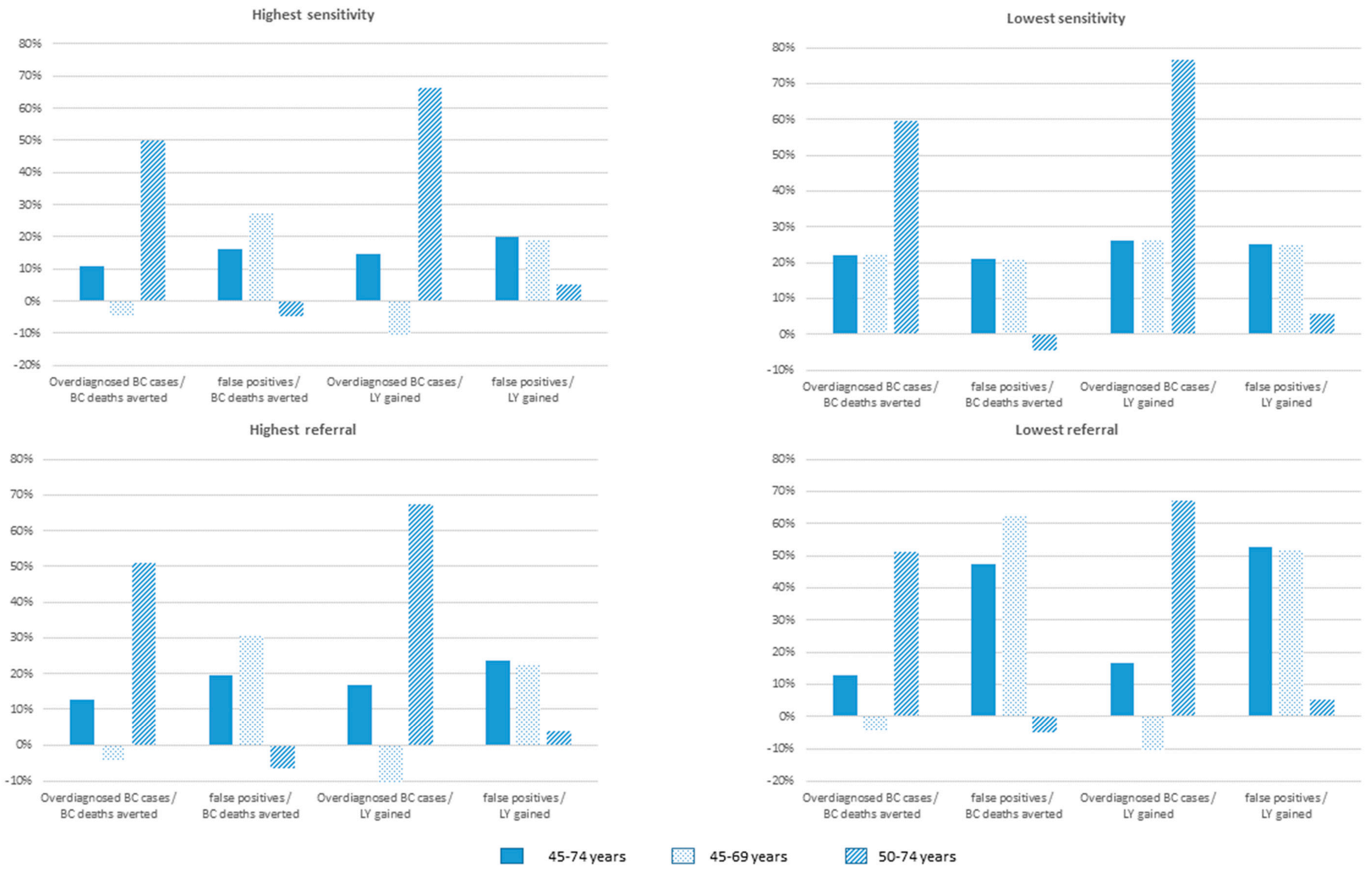
Highest sensitivity
Lowest sensitivity
Highest referral
Lowest referral
80%
70%
60%
50%
40%
30%
20%
10%
0%
-10%
-20%
Overdiagnosed BC cases / BC deaths averted
false positives / BC deaths averted
Overdiagnosed BC cases / LY gained
false positives / LY gained
45-74 years
45-69 years
50-74 years

Figure S14. *Cont.*

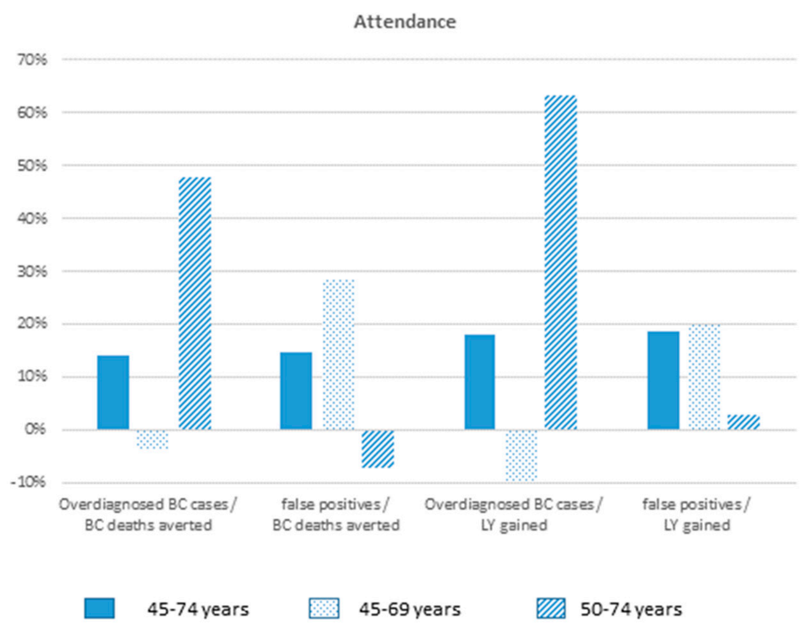

Figure S14. Percentage change in harms-to-benefit-ratios in comparison to the reference age-group 50–69, per screening scenario and varied parameter of the sensitivity analysis. FINLAND.

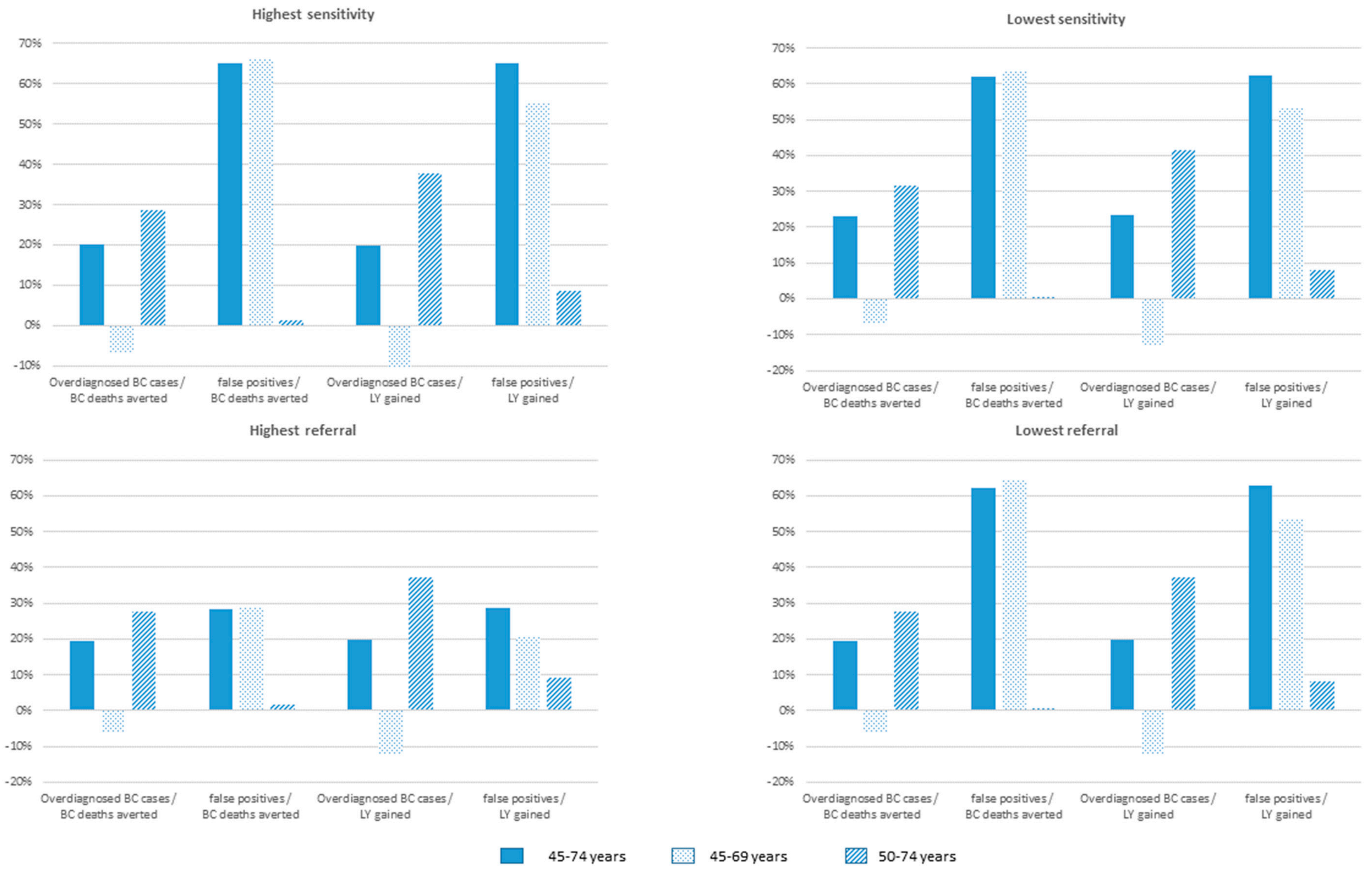

Highest sensitivity
Lowest sensitivity
Highest referral
Lowest referral
70%
60%
50%
40%
30%
20%
10%
0%
-10%
-20%
Overdiagnosed BC cases / BC deaths averted
false positives / BC deaths averted
Overdiagnosed BC cases / LY gained
false positives / LY gained
45-74 years
45-69 years
50-74 years

Figure S15. *Cont.*

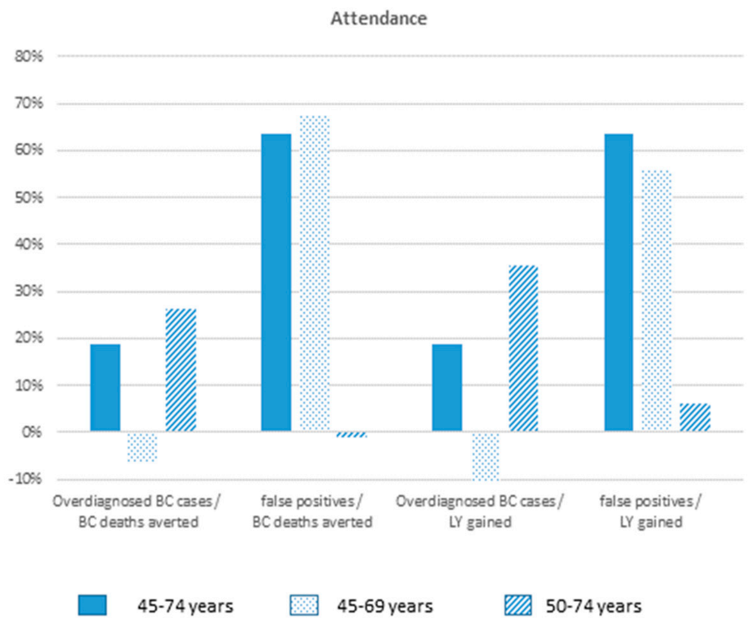

Figure S15. Percentage change in harms-to-benefit-ratios in comparison to the reference age-group 50–69, per screening scenario and varied parameter of the sensitivity analysis. THE NETHERLANDS.

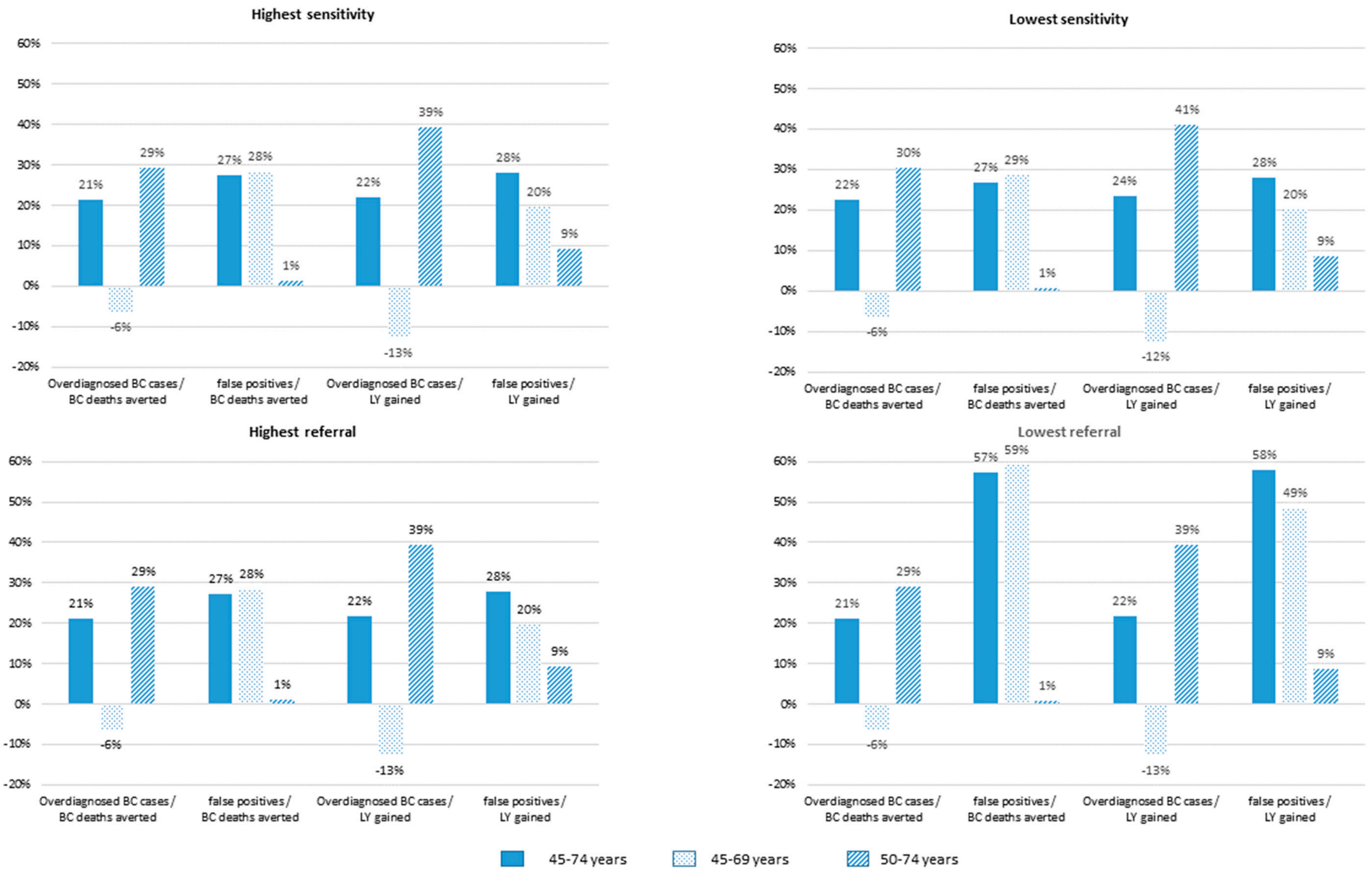

Highest sensitivity
Lowest sensitivity
Highest referral
Lowest referral
60%
50%
40%
30%
20%
10%
0%
-10%
-20%
Overdiagnosed BC cases / BC deaths averted
false positives / BC deaths averted
Overdiagnosed BC cases / LY gained
false positives / LY gained
21% -6% 29%
27% 28% 1%
22% -13% 39%
28% 20% 9%
22% -6% 30%
27% 29% 1%
24% -12% 41%
28% 20% 9%
21% -6% 29%
27% 28% 1%
22% -13% 39%
28% 20% 9%
21% -6% 29%
57% 59% 1%
22% -13% 39%
58% 49% 9%
45-74 years
45-69 years
50-74 years

Figure S16. *Cont.*

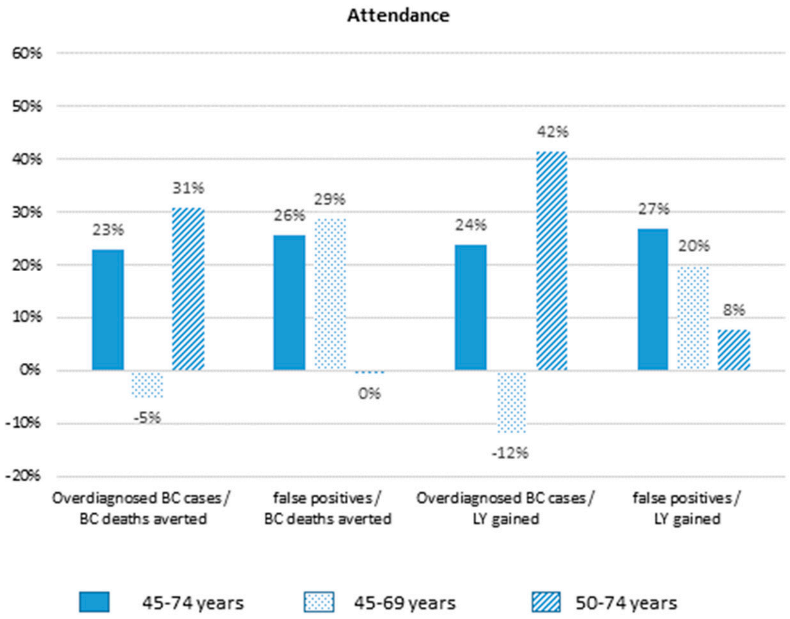

Figure S16. Percentage change in harms-to-benefit-ratios in comparison to the reference age-group 50–69, per screening scenario and varied parameter of the sensitivity analysis. ITALY.

References

1. Bjurstam, N.; Björneld, L.; Warwick, J.; Sala, E.; Duffy, S.W.; Nyström, L.; Walker, N.; Cahlin, E.; Eriksson, O.; Hafström, L.-O.; et al. The Gothenburg breast screening trial. *Cancer* **2003**, *97*, 2387–2396.
2. Tabar, L.; Vitak, B.; Chen, H.H.; Duffy, S.W.; Yen, M.F.; Chiang, C.F.; Krusemo, U.B.; Tot, T.; Smith, R.A. The Swedish two-county trial twenty years later—Updated mortality results and new insights from long-term follow-up. *Radiol. Clin. North. Am.* **2000**, *38*, 625-+.
3. Nyström, L.; Andersson, I.; Bjurstam, N.; Frisell, J.; Nordenskjöld, B.; Rutqvist, L.E. Long-term effects of mammography screening: Updated overview of the Swedish randomised trials. *Lancet* **2002**, *359*, 909–919.
4. de Koning, H.J.; Boer, R.; Warmerdam, P.G.; Beemsterboer, P.M.; van der Maas, P.J. Quantitative interpretation of age-specific mortality reductions from the Swedish breast cancer-screening trials. *J. Natl Cancer Inst.* **1995**, **87**, 1217–1223.
5. van Oortmarssen, G.J.; Habbema, J.D.; van der Maas, P.J.; de Koning, H.J.; Collette, H.J.; Verbeek, A.L.; Geerts, A.T.; Lubbe, K.T. A model for breast cancer screening. *Cancer* **1990**, *66,* 1601–1612.
6. Sankatsing, V.D.; Heijnsdijk, E.A.; van Luijt, P.A.; van Ravesteyn, N.T.; Fracheboud, J.; de Koning,H.J. Cost-effectiveness of digital mammography screening before the age of 50 in The Netherlands. *Int. J. Cancer* **2015**, *137*, 1990–1999.
7. Heinavaara, S.; Sarkeala, T.; Anttila, A. Impact of organised mammography screening on breast cancer mortality in a case-control and cohort study. *Br. J. Cancer* **2016,** *114*, 1038–1044.
8. Anttila, A.; Sarkeala, T.; Hakulinen, T.; Heinävaara, S. Impacts of the Finnish service screening programme on breast cancer rates. *BMC Public Health* **2008**, *8*.
9. Koleva-Kolarova, R.G.; Zhan, Z.; Greuter, M.J.W.; Feenstra, T.L.; De Bock, G.H. Simulation models in population breast cancer screening: A systematic review. *Breast* **2015**, *24*(4), 354–363.
10. Eddy, D.M.; Hollingworth, W.; Caro, J.J.; Tsevat, J.; McDonald, K.M.; Wong, J.B.; ISPOR-SMDM Modeling Good Research Practices Task Force. Model transparency and validation: a report of the ISPOR-SMDM Modeling Good Research Practices Task Force-7. *Med. Decis. Making* **2012**, *32*(5), 733–743.
11. Zielonke, N.; Gini, A.; Jansen, E.; Anttila, A.; Segnan, N.; Ponti, A.; Veerus, P.; de Koning, H.J.; van Ravesteyn, N.T.; Heijnsdijk, E.A.M.; et al. Evidence for reducing cancer specific mortality due to screening for breast cancer in Europe: a systematic review. *Eur. J. Cancer* **2020**, *127*, 191–206.
12. Puliti, D.; Miccinesi, G.; Collina, N.; De Lisi, V.; Federico, M.; Ferretti, S.; Finarelli, A.C.; Foca, F.; Mangode, L.; Naldoni, C.; et al. Effectiveness of service screening: A case-control study to assess breast cancer mortality reduction. *Br. J. Cancer* **2008**, *99*, 423–427.
13. Paap, E.; Verbeek, A.L.M.; Botterweck, A.A.M.; van Doorne-Nagtegaal, H.J.; Imhof-Tas, M.; de Koning, H.J.; Otto, S.J.; de Munck, L.; van der Steen, A.; Holland, R.; et al. Breast cancer screening halves the risk of breast cancer death: A case-referent study. *Breast* **2014**, *23*, 439–444.
